# Effect of Commercial Off-The-Shelf MAPS on γ-Ray Ionizing Radiation Response to Different Integration Times and Gains

**DOI:** 10.3390/s19224950

**Published:** 2019-11-13

**Authors:** Shoulong Xu, Jaap Velthuis, Qifan Wu, Yongchao Han, Kuicheng Lin, Lana Beck, Shuliang Zou, Yantao Qu, Zengyan Li

**Affiliations:** 1 Department of Engineering Physics, Tsinghua University, Beijing 100084, China; 2 School of resource Environment and Safety Engineering, University of South China, Hengyang 421001, China; zousl2013@126.com; 3 School of Physics, University of Bristol, Bristol BS8 1QU, UK; Jaap.Velthuis@bristol.ac.uk (J.V.); lana.beck@bristol.ac.uk (L.B.); 4 School of Nuclear Science and Engineering, University of South China, Hengyang 421001, China; 5 China Institute of Atomic Energy, Beijing 102413, China; quyantao@ciae.ac.cn; 6 Instituted of materials, China academy of engineering physics, Mianyang 621700, China; linkc06@caep.cn; 7 Northwest Institute of Nuclear Technology, Xi’an 710024, China; Lizy_THU@163.com

**Keywords:** COTS commercial MAPS, radiation response, integral time, gain

## Abstract

We report the γ-ray ionizing radiation response of commercial off-the-shelf (COTS) monolithic active-pixel sensors (MAPS) with different integration times and gains. The distribution of the eight-bit two-dimensional matrix of MAPS output frame images was studied for different parameter settings and dose rates. We present the first results of the effects of these parameters on the response of the sensor and establish a linear relationship between the average response signal and radiation dose rate in the high-dose rate range. The results show that the distribution curves can be separated into three ranges. The first range is from 0 to 24, which generates the first significant low signal peak. The second range is from 25 to 250, which shows a smooth gradient change with different integration times, gains, and dose rates. The third range is from 251 to 255, where a final peak appears, which has a relationship with integral time, gain, and dose rate. The mean pixel value shows a linear dependence on the radiation dose rate, albeit with different calibration constants depending on the integration time and gain. Hence, MAPS can be used as a radiation monitoring device with good precision.

## 1. Introduction

In a nuclear accident or in a strong radiation field, the detection of high dose-rate radiation and wide-range γ-rays is expensive and inefficient [1]. The use of commercial off-the-shelf (COTS) complementary metal oxide semiconductor (CMOS) monolithic active-pixel sensors (MAPS) as a γ-ray radiation detector has been reported. Martín Pérez et al. observed that the radiation response characteristic can be used for radiometric imaging [2], and that MAPS can be used to classify particles and the sensor is sensitive to soft X-rays [3]. Galimberti and Wang reported successful radiation detection using commercial off-the-shelf MAPS [4]. Ma et al. used Advanced RISC Machine (ARM) microcontrollers and ZigBee modules in combination with MAPS to detect low-energy radiation [5,6]. Arbor et al. reported a linear relationship between the MAPS radiation response signal, and the dose rate of γ-ray radiation field was proven [7]. Early reports on the application feasibility of using smartphones as a radiation measure detector have been published [8], Wei et al. also reported using mobile phones with MAPS cameras for radiation detection, which confirmed that after calibration, smartphones can be used as γ-ray measuring devices and for radiation safety control of high-level radioactive sources such as industrial radiography, γ-ray irradiation facilities, and medical treatment [9,10,11]. Another report focused on determining the heavy particle effect using an active-pixel sensor, which produced a significant radiation response from a single event [12]. However, few papers report the effect of setting the parameters of a MAPS video surveillance camera on the radiation response signal in the strong γ-ray radiation range. According to the MAPS working process, the integral time and gain of the set parameters most directly affect the radiation response signal.

In this study, we examined the distribution of eight-bit two-dimensional matrix of the MAPS output frame image using different setting parameters and dose rates. The image data are expressed in the form of a distribution curve. The abscissa is the pixel value, and the ordinate is the count of pixels of the pixel value in the image. We present the first result of the effect of different parameter values on the response signal and the linear relationship between the statistical value of the response signal and radiation dose rate in the high-dose rate range.

The rest of this paper is organized as follows: The experimental setup and the data processing methods are detailed in Section 2. The experimental results and the data processing are described in Section 3. Our conclusions are presented in Section 4.

## 2. Experiments

### 2.1. Initial Parameters of Image Sensors

**Figure 1:**
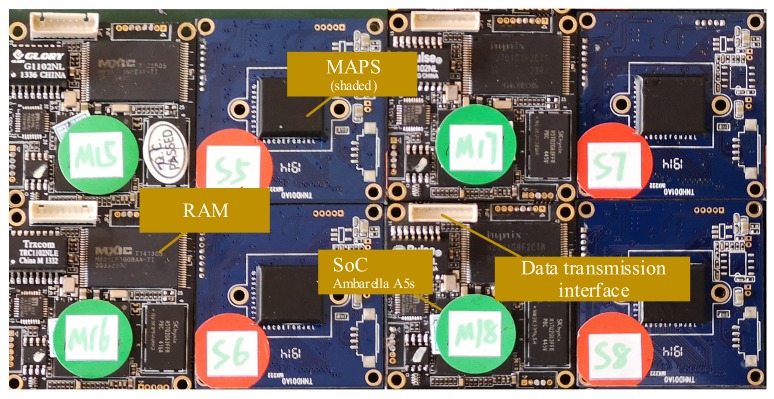
Picture of the monolithic active-pixel sensors (MAPS) module.

In the experiments, we used COTS CMOS MAPS (IMX 322 LQJ, Sony Corporation, Tokyo, Japan, ) with 6.4 mm pixel-type and approximately 2.43 M effective pixels, which are low cost (about USD $35), have low background noise, and are easy to obtain. The sensor provides an 8-bit response for each pixel. The chips are operated with 2.7 V, 1.2 V digital, and 1.8 V interface triple power supplies. All the COTS sensors were shaded with a layer of opaque plastic material and placed inside a homemade dark box to prevent any interference from visible light and to maintain constant room temperature during video recording. The test sensor was employed with no lenses and was operated in monochromatic mode with a rolling shutter. The integration time of the sensor is adjustable from 1/25 to 1/10,000 s. The parameter settings of the sensors were controlled by software, and video was recorded at a frame rate of 25 frames per second (fps) in a video format and streamed to hard disk. The integration time and gain of the sensors were manually adjusted; all the autoregulation and noise reduction functions and the white balance were turned off. Based on previous research results, radiation damage can be ignored when the radiation dose is less than 30 Gy [13] for the MAPS with a 4 T structure used in this study. The background noise with pixel values between 1 and 5 is the most common before irradiation, and the number of pixels with values greater than 10 is less than 0.2% of the total. The picture of the MAPS module samples is shown in Figure 1.

### 2.2. Experimental Setup

**Figure 2:**
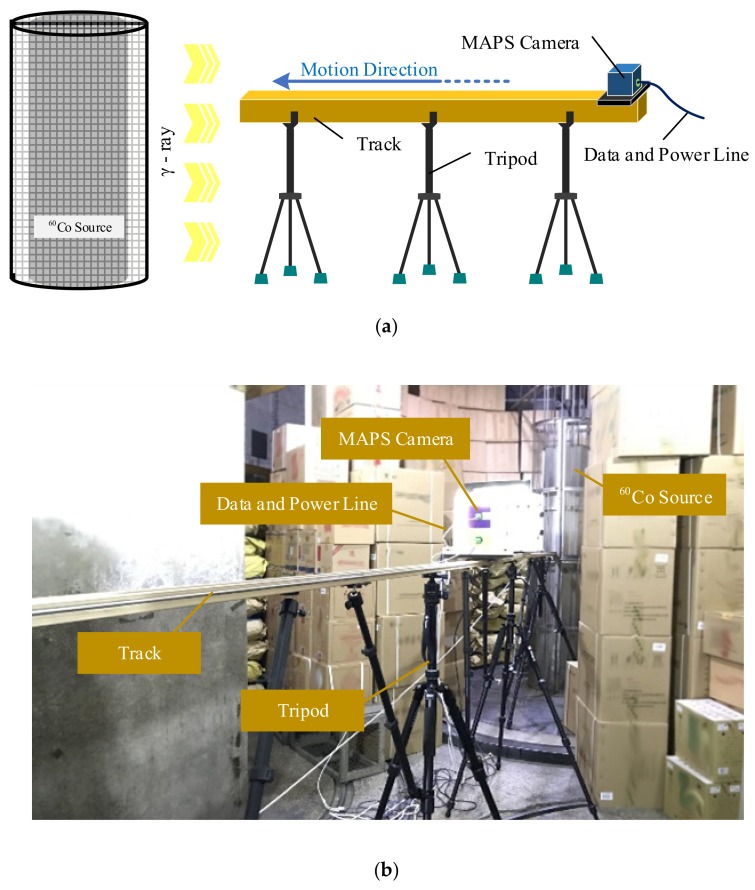
Experimental setup: (**a**) Schematic view of the experimental system and (**b**) picture of the experimental setup.

A cylindrical ^60^Co source from the China Institute of Atomic Energy (CIAE, Beijing, China) was used in all experiments. ^60^Co provides γ-rays of 1.17 and 1.33 MeV. The average activity of the source was 130 kCi, whereas the radiation non-uniformity was less than 5%. The ambient temperature during sensor testing was 20 °C. The CMOS MAPS sample sensors were irradiated with dose rates ranging from 51.61 to 479.24 Gy/h, which were obtained from low to high dose rates using a movable slider. The dose rates at each point on the slide track were calibrated. The total ionizing dose was measured using a radiochromic film dosimeter, and the dose rate was calculated as the ratio of the total ionizing dose to the irradiation time. The experimental setup is shown in Figure 2. The test sensors were operated nearly continuously during all experiments. Signal data were transmitted using a 4800LX cable, and the maximum video bitrate was 13 Mbps. Video files were recorded at 25 fps by a computer during all experiments, and the data were imported using MATLABR 2014a (Math Works Inc., Natick, MA, USA) and then split into individual frames. During experiments, the systems controlling aperture, shutter, gain, and white balance were set to manual, and noise reduction functions and exposure compensation functions were turned off. All the data were collected before the dose rate reached 30 Gy.

The recorded video data were processed on a PC by MATLAB. Each frame of the video was transformed into an 8-bit gray value matrix for analysis. The 8-bit gray value is the sensor in analog-to-digital converter (ADC) units, and the range of gray value was from 0 to 255. To obtain more accurate statistics, radiation response events in 100 consecutive frames were counted together. The mean pixel value (*S_k_*) of the selected images at the radiation dose rate of *k* was calculated as follows:

(1) $$S_{k}=\frac{1}{MN}\overset{j=M}{\underset{j=1}{\sum }}\overset{i=N}{\underset{i=1}{\sum }}\left(E_{i,j}-I_{i,j}\right)$$

where *I_i,j_* is the pixel value of the *i*th pixel in the *j*th frame before irradiation, *E_i,j_* is the pixel value of the *i*th pixel in the *j*th frame at the radiation dose rate of *k*, *M* is the number of frames, and *N* is the pixel count.

## 3. Discussion

**Figure 3:**
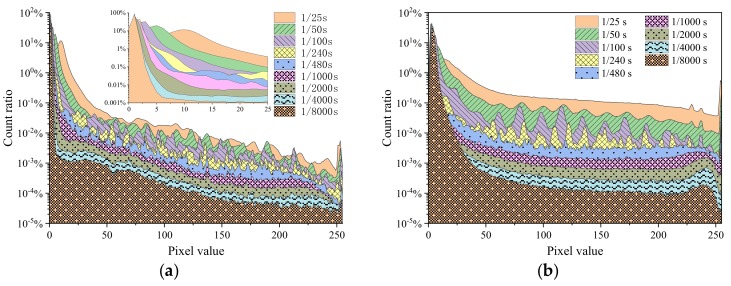
Distribution of the count fraction of pixels at a dose rate of 51.61 Gy/h and (**a**) a gain of 6 dB and (**b**) a gain of 42 dB in frames captured at using integration times ranging from 1/8000 to 1/25 s.

Figure 3 shows the distribution of the count fraction of pixel signals at a dose rate of 51.61 Gy/h and using two gains, 6 and 42 dB, in frames captured with integration times ranging from 1/8000 to 1/25 s. For a gain of 6 dB (Figure 3a), the first peak corresponds to gray levels below 15. The position of peak shifts to larger pixel values with increasing of integration time, which indicates that more pixels yield stronger pixel signals due to exposure to more photons. However, no obvious change in the position of peak for the larger gain of 42 dB (Figure 3b) was observed. For both amplifications, the height of the pixel value distributions between 75 and 200 followed the integration time; longer integration times yield higher count fraction distributions. The count and maximum value of peaks in that range increased with larger integration time, and curves at 42 dB were smoother than those at 6 dB with the exception of integration times of 1/100 and 1/240 s. This indicates that the distribution for lower integration times and larger gains is smoother, which indicates that a quantization issue exists in the sensor. We noticed that a significant peak exists in the range larger than 250; the peak is narrower and smaller at the larger gain of 42 dB. The shape of radiation response events has been reported [13], and this peak might be caused by some saturation and supersaturation radiation response events in frames.

**Figure 4:**
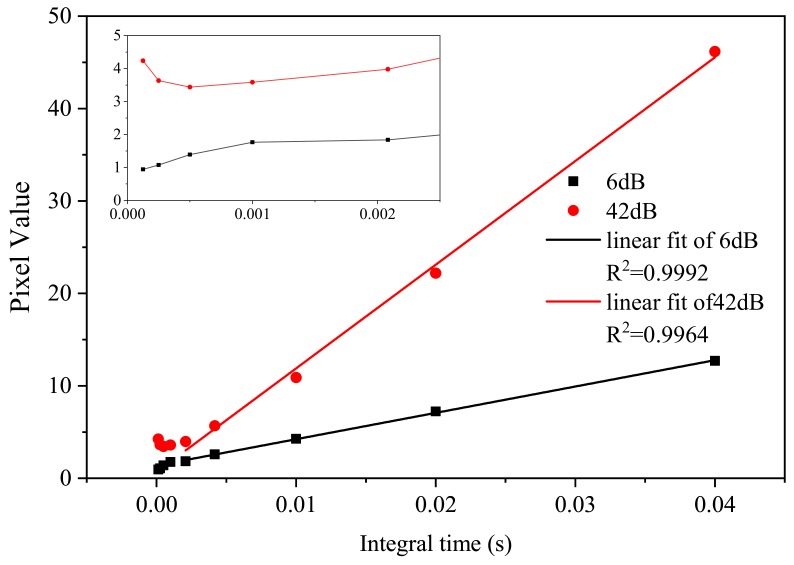
Average pixel value as a function of the integration time for a gain of 6 and 42 dB during irradiation at 51.61 Gy/h.

We calculated the average pixel value in Figure 3 in the range between 75 to 250 for each integration time. The relationship between the average pixel value and integration time at the irradiation dose rate of 51.61 Gy/h is plotted in Figure 4. Figure 4 shows that for integration times larger than 1/480 s, a linear relationship exists between the average pixel value and integration time. The linearity for 6 dB is better than that of 42 dB.

**Figure 5:**
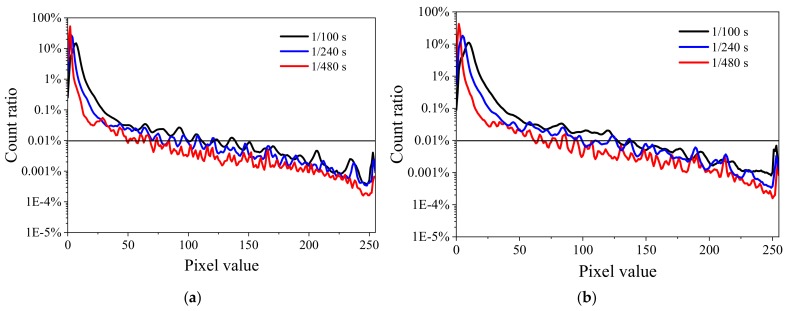

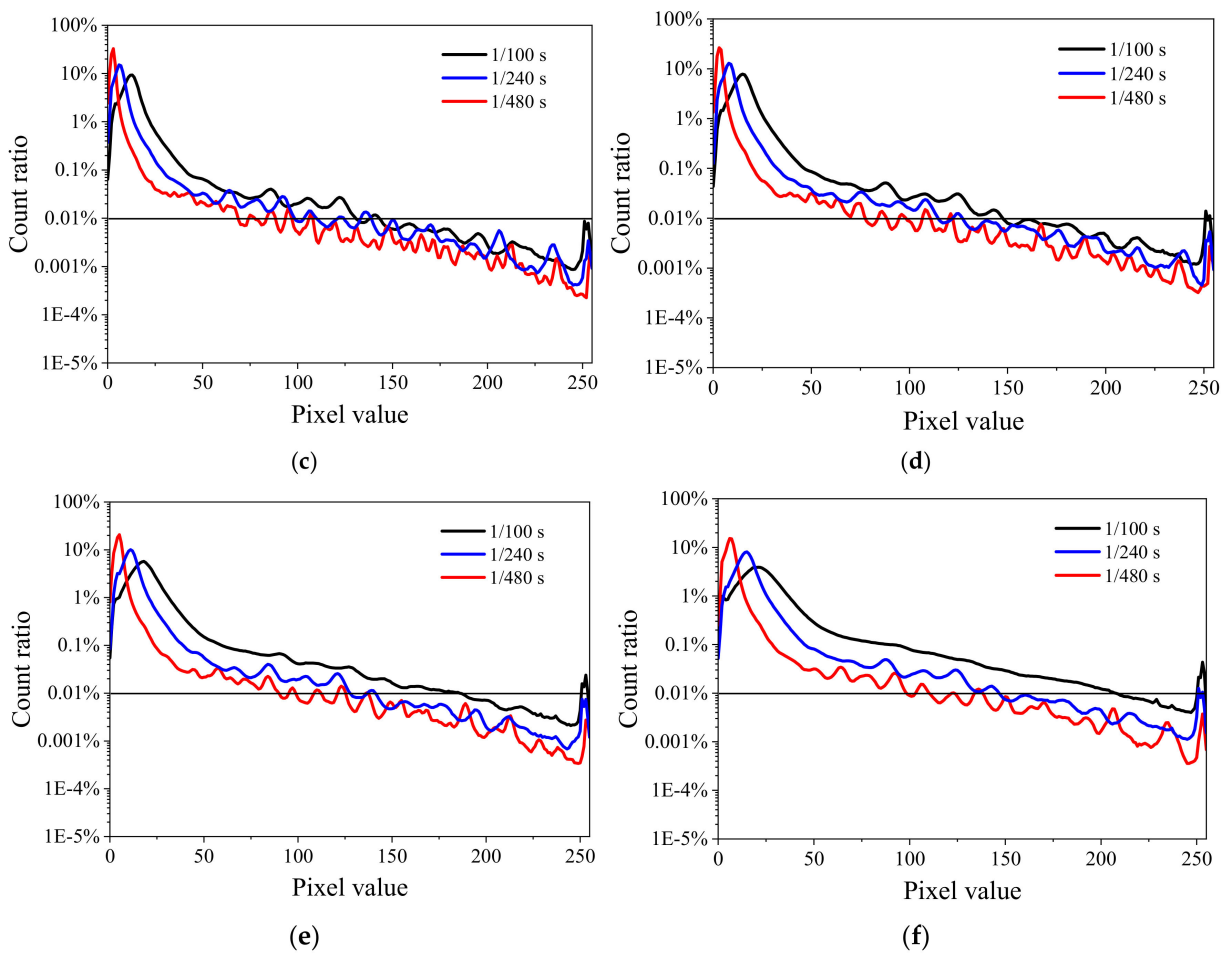
Distribution of the count fractions of pixels at a gain of 6 dB for six different dose rates in frames captured at integration times of 1/100, 1/240, and 1/480 s: (**a**) 64.48, (**b**) 95.00, (**c**) 119.5, (**d**) 153.41, (**e**) 200.32, and (**f**) 265.22 Gy/h.

The distribution of count fractions of pixels at a gain of 6 dB and at six irradiation dose rates from 64.48 to 265.22 Gy/h at three integration times of 1/100, 1/240, 1/480 s are shown in Figure 5. The pixel value was calculated using Equation (1). As the irradiation dose rate increases, the maximum value increases, and the number of pixels with values larger than 25 increases. A peak for pixel signals larger than 250 occurred at irradiation doses rate larger than 200 Gy/h. This peak occurred at all measured integration times.

**Figure 6:**
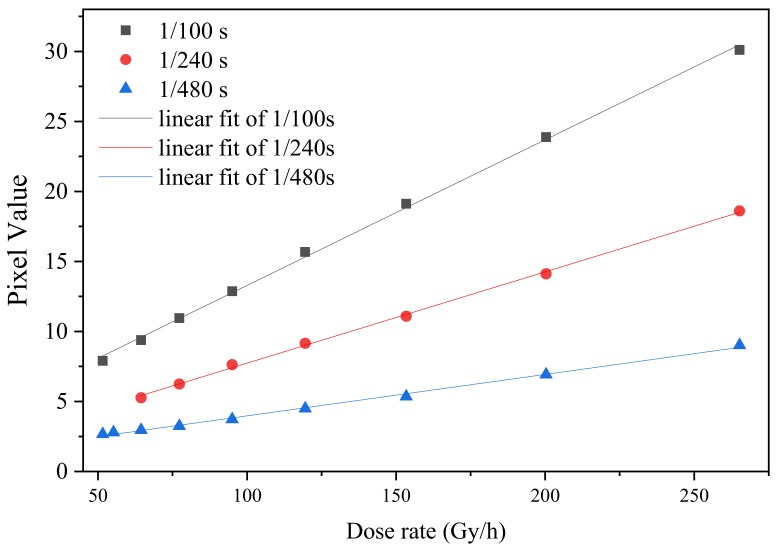
Average pixel signal as a function of irradiation dose rate for integration times of 1/100, 1/240, and 1/480 s at a gain of 6 dB.

We calculated the average pixel signal in Figure 5 in the range between 25 to 250, i.e., excluding the peak around 250. The relationship between mean pixel value and dose rate at the integration times of 1/100, 1/240, and 1/480 s is shown in Figure 6. The linearities of the fit of 1/100, 1/240, and 1/480 s are 0.9985, 0.9986, and 0.9964, respectively.

**Figure 7:**
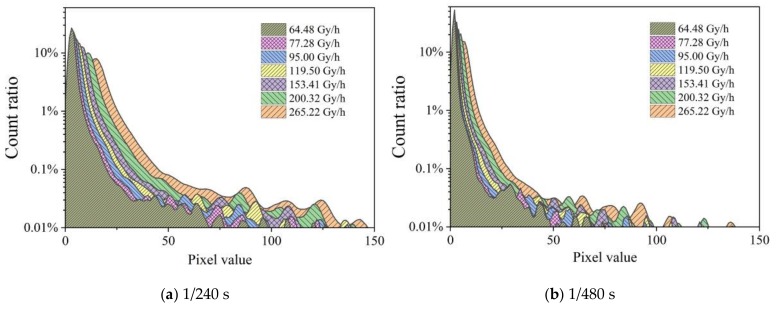
Distribution of the count fraction of pixels at a gain of 6 dB and for two integration times captured at dose rates ranging from 64.48 to 265.22 Gy/h.

Figure 7 shows the distribution of count fractions of pixels at a gain of 6 dB and integration times of 1/240 and 1/480 s at dose rates ranging from 64.48 to 265.22 Gy/h. The pixel value was calculated using Equation (1). The position of the peak shifts to larger pixel values with increasing dose rates as more pixels receive hits and even multiple hits. This is particularly clear for the first peak, i.e., the peak with values below 25. Figure 7 compares the effects of dose rate changes on the distribution under different integration times. The peak locations of these curves in the range larger than 25 also depend on the integration time. The same peak structure was observed for both integration times, but the peak locations move with integration time.

**Figure 8:**
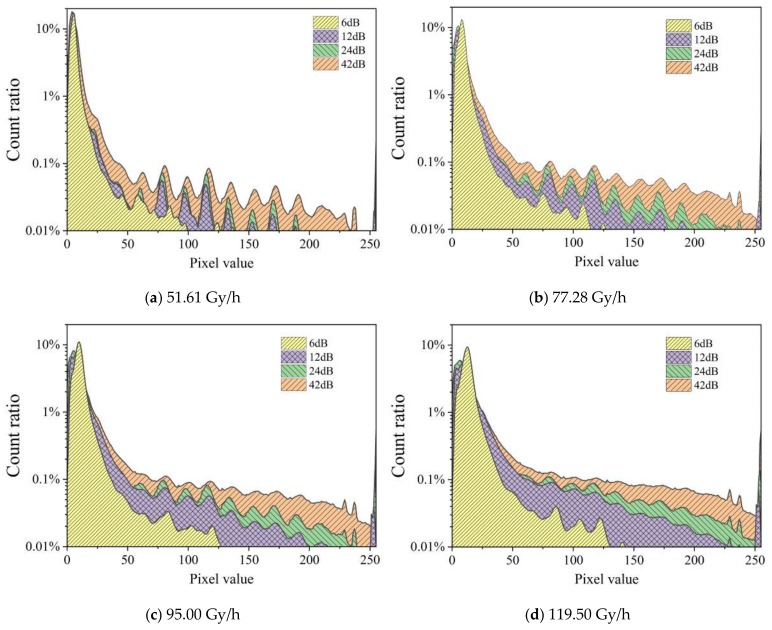
Distribution of the count fraction of pixels for an integration time of 1/100 s at four dose rates captured using gains of 6, 12, 24, and 42 dB.

Figure 8 shows the distribution of the count fraction of pixels at an integration time of 1/100 s at four dose rates captured using gains of 6, 12, 24, and 42 dB. The figure compares the effect of adjusting the gain on the statistical curve under different dose rates. The graphs show that for higher gains, more pixels have higher signals, and higher radiation dose rates yield higher pixel values. All graphs display peaks in the same location irrespective of the gain. This indicates issues with the sensor.

**Figure 9:**
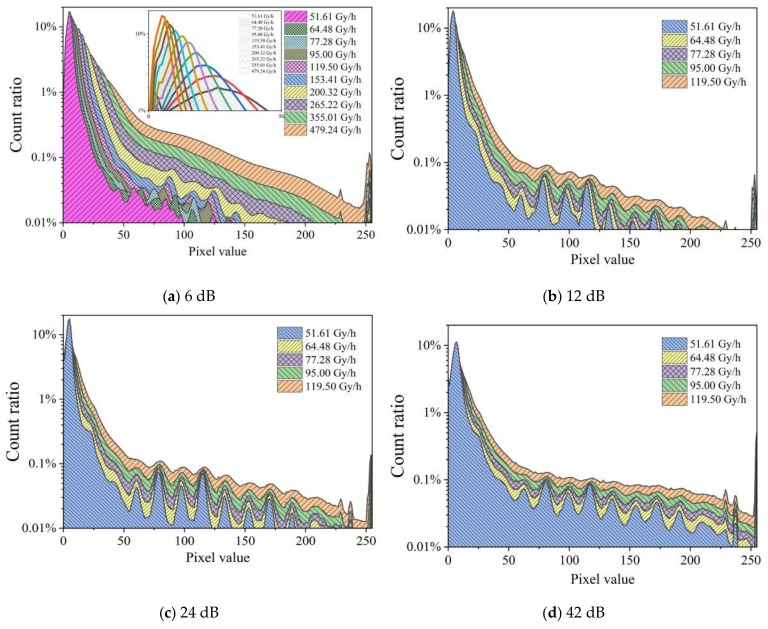
Distribution of the count fraction of pixels at an integration time of 1/100 s for four values of the gain captured at dose rates ranging from 51.61 to 119.50 Gy/h (from **b** to **d**), and from 51.61 to 479.24 Gy/h (**a**).

Figure 9 shows the distribution of the count fraction of pixels at an integral time of 1/100 s captured at different dose rates ranging from 51.61 to 119.50 Gy/h, and measured using gains of 12, 24, and 48 dB using dose rates ranging from 51.61 to 479.24. For 6 dB, we observed that the peak at low pixel values moves to the right with increasing dose rate. However, this was not observed for any of the higher gains. Figure 9 shows that larger gains result in more pixels with larger values. Peaks are generated at the same positions for gains from 12 dB. This indicates a quantization issue in the data as the same photons should yield higher signals at higher gains.

In summary, we can control the distribution range of pixel values between 0 and 255 by adjusting the integration time or gain and we can separate the distribution curves into three ranges. The first range is 0 to 24, which incorporates the first significant peak. The second range is 25 to 250, which shows a smooth gradient change with different integration times, gains, and dose rates. The last range is 251 to 255, where a peak occurs that is related to integration time, gain, and dose rate. Since the γ-ray dose rate detection relies on the pixel values of frames, studying the response signals is crucial. The results show that a more stable response is obtained for larger gains and lower integral times. However, a lower integral time means less sampling efficiency of the radiation response signal and a smaller dynamic range, which is an important factor affecting the detection accuracy and efficiency.

**Figure 10:**
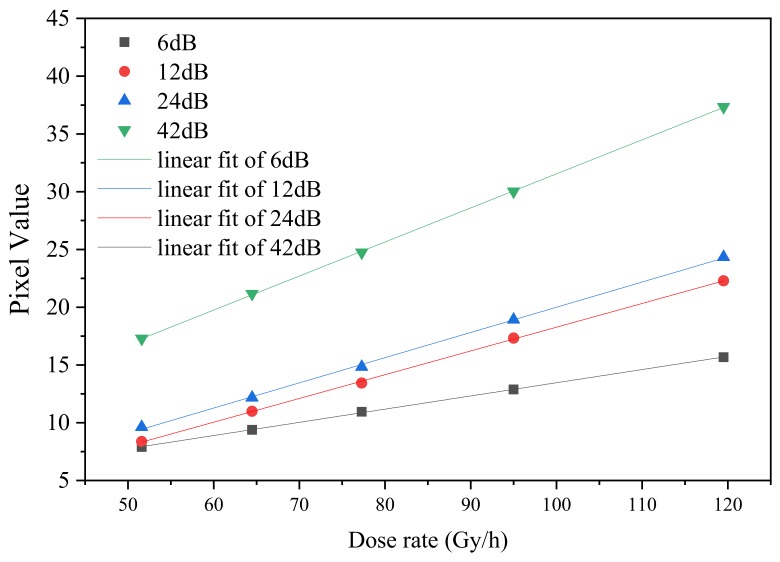
Mean pixel value as a function of the irradiation dose rate for gains of 6, 12, 24, and 42 dB at an integration time of 1/25 s.

Figure 10 shows the dependence of the mean pixel signal on the irradiation dose rate for gains of 6, 12, 24, and 42 dB at an integration time of 1/25 s, where only pixels with signals ranging between 25 to 250 in Figure 9 are included. The pixel value was calculated using Equation (1). The linearity of the linear fit of 6, 12, 24, and 42 dB are 0.9997, 0.9996, 0.9990, and 0.9998, respectively, demonstrating good linearity.

## 4. Conclusions

In this work, we investigated the effect of integration time and gain of COTS MAPS on ionizing radiation detection. We discussed the potential use of MAPS at a high dose rate and wide range γ radiation detector. The pixel response distribution range of pixel value can be controlled by adjusting the integration time or gain from 50 to 265 Gy/h. The distribution curves can be separated into three ranges. The first range is 0 to 24, which generates the first significant peak. The second range is from 25 to 250, which shows a smooth gradient change with different integration times, gains, and dose rates. The last range is 251 to 255, which might be caused by some saturation and supersaturation radiation response events in the frames. This peak occurs for all measured integration times at an irradiation dose rate ranging from 51.61 to 265.22 Gy/h. More pixels with larger pixel values are generated with larger dose rates, which shifts the position of peak to larger pixel values. The mean pixel value shows a linear dependence on the radiation dose rate, albeit with different calibration constants depending on the integration time and gain. Hence, MAPS can be used with good precision as a radiation monitoring device under different settings for different doses.
